# Activity of dalotuzumab, a selective anti-IGF1R antibody, in combination with erlotinib in unselected patients with Non-small-cell lung cancer: a phase I/II randomized trial

**DOI:** 10.1186/2162-3619-3-26

**Published:** 2014-11-07

**Authors:** Teresa Moran, Enriqueta Felip, Vicki Keedy, Hossein Borghaei, Frances A Shepherd, Amelia Insa, Holly Brown, Timothy Fitzgerald, Sriram Sathyanarayanan, John F Reilly, David Mauro, Karl Hsu, Li Yan, David H Johnson

**Affiliations:** 1 Catalan Institut of Oncology-Badalona, Hospital Universitari Germans Trias i Pujol and Universitat Autònoma de Barcelona, Barcelona, Spain; 2 Vall d’Hebron University Hospital, Barcelona, Spain; 3 Vanderbilt University Medical Center, Nashville, TN USA; 4 Fox Chase Cancer Center, Philadelphia, PA USA; 5 University Health Network Princess Margaret Cancer Centre, University of Toronto, Ontario, Canada; 6 Hospital Clínico Universitario de Valencia, Valencia, Spain; 7 Merck & Co., Inc., Whitehouse Station, NJ USA; 8 Sanofi Aventis, Bridgewater, NJ USA; 9 UT Southwestern University, Dallas, TX USA

**Keywords:** Non-small-cell lung cancer, Epidermal growth factor receptor, Insulin growth factor receptor, Dalotuzumab, Phase I/II trial

## Abstract

**Background:**

We investigated the safety and antitumor activity of dalotuzumab, a selective anti-insulin growth factor 1 receptor monoclonal antibody (IGF1R MoAb), plus erlotinib in a sequential phase I/II trial in unselected patients with refractory advanced non-small-cell lung cancer (NSCLC).The phase I trial determined the recommended dose and safety of erlotinib plus dalotuzumab at 5 mg/kg or 10 mg/kg weekly in 20 patients. The phase II trial compared outcomes to erlotinib alone and erlotinib plus dalotuzumab at the mg/kg established in the phase I trial.

**Results:**

Erlotinib at 150 mg plus dalotuzumab at 10 mg/kg was safe. The phase II trial included 37 patients in the erlotinib arm and 38 patients in the erlotinib plus dalotuzumab arm. Progression-free survival was 1.6 versus 2.5 months, overall survival was 10.2 and 6.6 months, and the objective response rate was 7.9% and 2.7%, respectively, with no significant differences between the two arms. Grade 3-5 adverse events occurred in 11 (28.9%) versus 13 (35.1%) patients, respectively. The most frequent adverse events were asthenia (36.8% vs. 37.8%), dehydration (5.3% vs. 2.7%), diarrhea (71% vs. 81.1%), hyperglycemia (13.1% vs.18.9%), and skin-related toxicities (92.1% vs. 86.4%).

**Conclusion:**

The addition of dalotuzumab to erlotinib did not improve efficacy outcome in patients with refractory advanced NSCLC.

## Introduction

Insulin-like growth factor 1 receptor (IGF1R) is overexpressed in a wide variety of human malignances, including non-small-cell lung cancer (NSCLC) [1–3]. Additionally, high co-expression of IGF1R and epidermal growth factor receptor (EGFR) has been correlated with shorter disease-free survival in NSCLC patients [4]. Cross-talk between the EGFR and IGF1R pathways contributes to transformation, growth, and tumor responsiveness to EGFR inhibitors [5].

Dalotuzumab, formerly MK-0646, is a humanized IgG1 anti-IGF1R monoclonal antibody (MoAb) that selectively binds to IGF1R without binding to insulin receptor (IR). By binding to the extracellular domain of the receptor, dalotuzumab blocks ligand binding and inhibits receptor autophosphorylation by up to 90%, leading to a block in IGF1- and IGF2-mediated cell proliferation *in vitro* and down-regulation of cell surface receptors by 75% to 90%. According to previous phase I trials, dalotuzumab reached a biologically optimal concentration when a dose of 10 mg/kg/week was administered and plasma IGF1R levels increased after dalotuzumab administration independently of the administered dose [6, 7].

We hypothesized that dual inhibition of the EGFR and IGF1R pathways could prove beneficial in NSCLC patients. We therefore performed a phase I/II trial testing the combination of erlotinib and dalotuzumab in unselected, advanced NSCLC patients who were refractory to previous chemotherapy.

## Methods

### Patient selection

**Figure 1:**
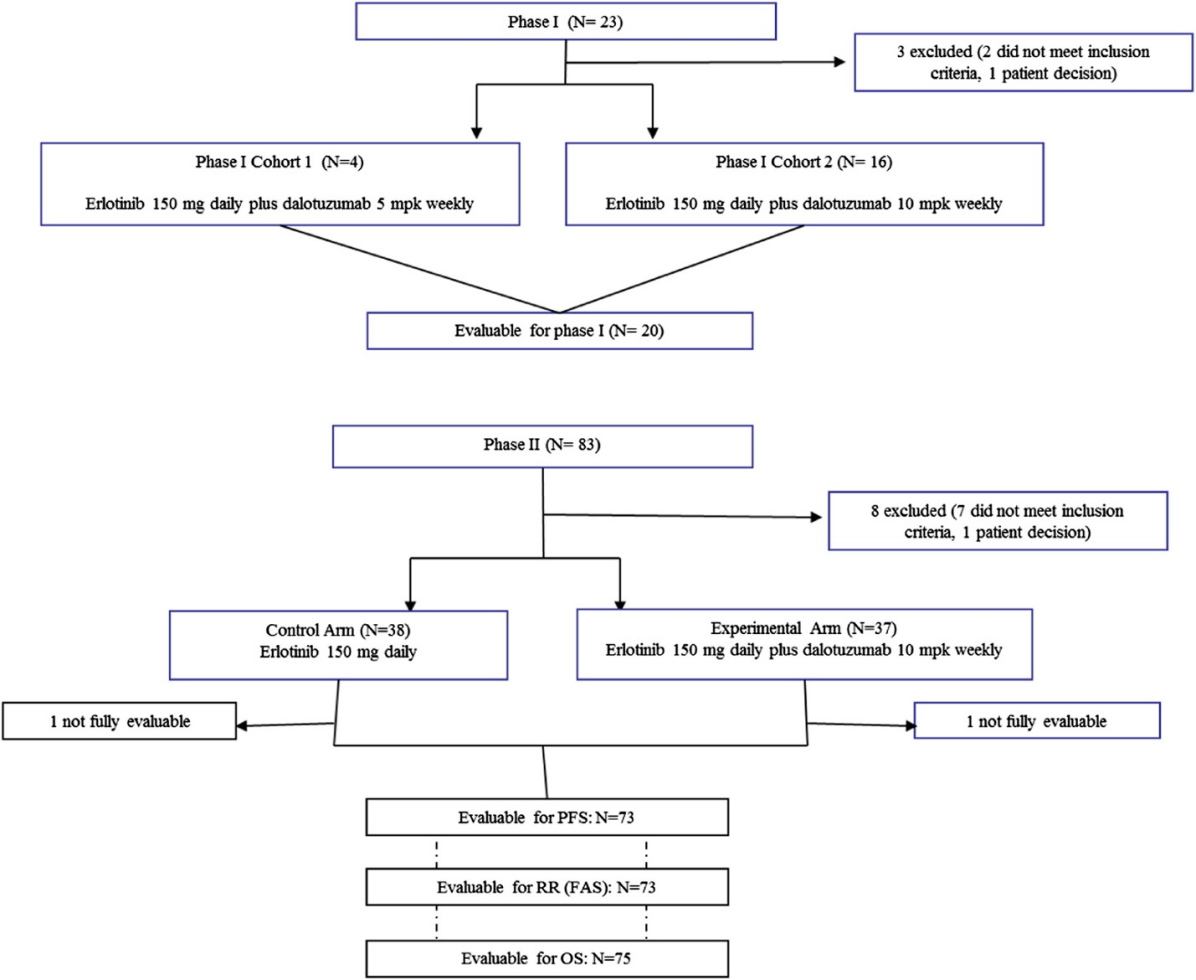
**CONSORT diagram showing patient disposition through the phase I and phase II trials.**

This study was designed as a phase I/II trial. Patients were recruited from five centers for the phase I trial and 20 centers for the phase II trial from Europe, the United States and Canada (Figure 1). Patients were eligible for inclusion if they were 18 years or older and had histologically documented advanced NSCLC refractory to previous therapy (at least one and no more than two previous chemotherapy regimens) The study was conducted in accordance with the Declaration of Helsinki and Good Clinical Practice Guidelines. The protocol was approved by local ethics committees at each participating center, and all patients gave their signed informed consent for participation in the study.

### Study design and treatment

The phase I trial consisted of a safety and tolerability run-in study testing dalotuzumab at two dose levels that had previously been demonstrated as safe with an adequate pharmacodymamic profile (5 and 10 mg/kg). Erlotinib was administered as a daily oral dose of 150 mg and dalotuzumab as a 60-minute weekly intravenous infusion. Dalotuzumab was administered following a "3 + 6" escalation scheme design, with an anticipated intermediate dose level of 7.5 mg/kg in the event of intolerable toxicity at the higher dose level.

In the phase II trial, patients were randomized to the control or experimental arm. Patients in the control arm received erlotinib alone, and those in the experimental arm received erlotinib plus dalotuzumab at the dose level determined by the phase I trial. Both dalotuzumab and erlotinib were provided by the sponsor of the trial, Merck Sharp & Dohme Corp., a subsidiary of Merck & Co, Inc., Whitehouse Station, NJ, USA.

### Endpoints and statistical considerations

For the phase I trial, the primary objective was to determine the safety and tolerability of erlotinib in combination with dalotuzumab in patients with advanced NSCLC. For clinical assessment of toxicity, patients were evaluated weekly and adverse events were graded according to the National Cancer Institute Common Terminology Criteria for Adverse Events version 3.0 [8]. For the purpose of determination of protocol dose escalation in phase I, dose limiting toxicity (DLT) was defined based on the events occurring within the first 4 weeks of therapy. For patient management, dose modification would occur in the event of DLT occurring during any cycle of therapy. Hematologic DLT were defined as grade 4 neutropenia lasting for ≥7 days, grade 3 or 4 neutropenia with fever >38.5° and/or infection requiring antibiotics or anti-fungal therapy and grade 4 thrombocytopenia (≤25.0 × 10^9^/L). Non-hematologic DLT were defined as any ≥ grade 3 non-hematologic toxicity, with the specific exception of grade 3 skin toxicity, nausea and/or vomiting, diarrhea, dehydration or hyperglycemia that in the opinion of the investigator occurred in the setting of inadequate compliance with supportive care measures and lasted less than 48 hours.

For the phase II trial, the primary efficacy endpoint was progression-free survival (PFS); secondary endpoints were response rate, overall survival (OS) and safety and tolerability. PFS was defined as the time from randomization until either radiographic evidence of disease progression or death due to any cause, whichever occurred first. OS was defined as the time from randomization to death due to any cause. Response was graded according to the Response Evaluation Criteria in Solid Tumors (RECIST) 1.0 guidelines [9]. Radiographic evaluation was performed every six weeks during the first 48 weeks and every three months thereafter. For sample size calculation and PFS estimation, 49 PFS events in both control and experimental arms were planned and 68 patients with a projected follow-up of 5 months were needed. The present study had 80% power to detect an improvement in PFS defined as a 45.5% reduction in the hazard ratio in the combination arm. A 45.5% reduction in hazard rate corresponds to 1.84 months in improvement in median PFS compared to the erlotinib arm.

## Results

### Patients

From 19 Mar 2008 to 5 Jun 2009, 23 patients were enrolled in the phase I trial. Four patients were included in the first dose cohort (erlotinib 150 mg daily orally plus dalotuzumab 5 mg/kg/week intravenously) and sixteen in the second dose cohort (erlotinib 150 mg daily orally plus dalotuzumab 10 mg/kg/week intravenously). From 11 Aug 2010 to 24 Mar 2011, 83 patients were enrolled in the phase II trial. Eight were excluded from the analysis leaving 75 patients, 38 in the control arm and 37 in the experimental arm. Clinical characteristics for patients in both phases are summarized in Table 1. Although the phase I trial was based in a "3 + 6" escalation scheme design, five patients were removed due to progressive disease before the first four weeks of dosing that were needed to monitor for DLTs. Three additional patients were included by decision of the study sponsor to ensure that the final sample included at least 9 fully evaluable patients.

**Table 1:** **Baseline patient characteristics**

	Phase I			Phase II	
	Dalotuzumab 5 mg/kg plus erlotinib cohort 1	Dalotuzumab 10 mg/kg plus erlotinib cohort 2		Erlotinib	Dalotuzumab plus erlotinib
	n =4	n =16		n =38	n =37
**Gender**			**Gender**		
Male	4 (100%)	14 (87.6%)	Male	28 (73.7%)	27 (73%)
Female	0 (0%)	2 (12.5%)	Female	10 (26.3%)	10 (27%)
**Age (years)**			**Age (years)**		
Mean	53.8	61.9	Mean	58.5	61.9
SD	3.9	7.7	SD	10.4	7.83
Median	53.5	62.0	Median	59.0	62.0
Range	50 to 58	50 to 72	Range	36 to 80	45 to 77
**Race**			**Race**		
Caucasian	4 (100%)	16 (100%)	Caucasian	36 (94.7%)	37 (100%)
Asian	0 (0%)	0 (0%)	Asian	2 (5.3%)	0 (0%)
**ECOG performance status**			**ECOG performance status**		
0	4 (100%)	5 (31.4%)	0	13 (34.2%)	11 (29.7%)
1	0 (0%)	11 (68.8%)	1	24 (63.2%)	24 (64.9%)
2	0 (0%)	0 (0%)	2	1 (2.6%)	2 (5.5%)
**Stage**			**Stage**		
IIIB	1 (25%)	2 (12.5%)	IIIB	9 (23.7%)	4 (10.8%)
IV	3 (75%)	14 (87.5%)	IV	29 (76.3%)	33 (89.2%)
**Smoking history**			**Smoking history**		
Current smoker	1 (25%)	5 (31.1%)	Current smoker	7 (18.4%)	12 (32.4%)
Former smoker	3 (75%)	7 (43.8%)	Former smoker	20 (52.6%)	21 (56.8%)
Never smoker	0 (0%)	4 (25%)	Never smoker	11 (28.9%)	4 (10.8%)
**Previous therapies**			**Previous treatments**		
Mean	1.5	1.5	First-line only	20 (52.6%)	27 (72.9%)
Median	1	1.5	First- and second-line	18 (47.4%)	10 (27.02%)
Range	1 to 3	1 to 3	Prior platinum-containing regimen	38 (100%)	35 (94.6%)
**Previous diagnosis of diabetes**	0 (0%)	4 (25%)	**Previous diagnosis of diabetes**	4 (10.4%)	2 (5.4%)
**Previous treatments for diabetes**	0 (0%)	4 (25%)	**Previous treatments for diabetes**	4 (10.4%)	2 (5.4%)
			Glimepiridine	1 (2.6%)	0 (0%)
Glyburide	0 (0%)	1 (6.2%)	Glyburide	1 (2.6%)	0 (0%)
Insulin	0 (0%)	1 (6.2%)	Insulin	0 (0%)	1 (2.7%)
Metformin	0 (0%)	1 (6.2%)	Metformin	1 (2.6%)	1 (2.7%)
Rapaglinide	0 (0%)	1 (6.2%)	Pioglitazone	1 (2.6%)	0 (0%)
**Histology**			**Histology**		
Adenocarcinoma	1 (25%)	5 (31.3%)	Adenocarcinoma	15 (39.5%)	14 (37.8%)
Squamous cell carcinoma	3 (75%)	4 (25%)	Squamous cell carcinoma	6 (15.8%)	11 (29.7%)
Large cell carcinoma	0 (0%)	1 (6.3%)	Large cell carcinoma	0 (0%)	1 (2.7%)
Not otherwise specified	0 (0%)	6 (37.5%)	Not otherwise specified	17 (44.7%)	11 (29.7%)

ECOG = Eastern Cooperative Oncology Group.

### Safety

In the phase I trial, the most common toxicities were those related to erlotinib. Thirteen patients (65%) developed diarrhea (two grade 3), and 50% suffered ≤ grade 2 rash or acneiform dermatitis (Table 2). One patient experienced grade 3 erythema. Dalotuzumab-related toxicities include hyperglycemia in 8 patients (five grade 3). Four of these 8 patients had a previous diagnosis of diabetes mellitus and were receiving treatment for diabetes before initiating the study.

**Table 2:** **Summary of adverse events**

Event		Phase I		Phase II	
		Dalotuzumab 5mg/kg plus erlotinib	Dalotuzumab 10 mg/kg plus erlotinib	Erlotinib	Dalotuzumab 10 mg/kg plus erlotinib
		N = 4	N = 16	N = 38	N = 37
**Diarrhea**					
	AE	1 (25%)	12 (75%)	27 (71%)	30 (81.1%)
	SAE	0	2 (12.6%)	0	0
	Drug-related AE	1 (25%)	10 (62.5%)	18 (47.3%)	16 (43.2%)
	g3-5	0	2 (12.5%)	1 (2.6%)	3 (8.1%)
**Nausea**					
	AE	0	5 (31.2%)	17 (44.7%)	17 (45.9%)
	SAE	0	0	0	0
	Drug-related AE	0	3 (18.7%)	4 (10.5%)	6 (16.2%)
	g3-5	0	0	0	0
**Stomatitis**					
	AE	2 (50%)	5 (31.2%)	3 (7.9%)	4 (10.8%)
	SAE	0	0	0	0
	Drug-related AE	1 (25%)	3 (18.5%)	2 (5.3%)	3 (8.1%)
	g3-5	0	0	0	0
**Asthenia**					
	AE	1 (25%)	10 (62.5%)	14 (36.8%)	14 (37.8%)
	SAE	0	0	1 (2.6%)	0
	Drug-related AE	0	1 (6.2%)	7 (18.4%)	3 (8.1%)
	g3-5	0	1 (6.2%)	2 (5.3%)	2 (5.4%)
**Hepatobiliary disorders**					
	AE	0	0	4 (10.5%)	4 (10.8%)
	SAE	0	0	0	2 (5.4%)
	Drug-related AE	0	0	1 (2.6%)	0
	g3-5	0	0	0	2 (5.4%)
**Hyperglycemia**					
	AE	0	8 (50%)	5 (13.1%)	7 (18.9%)
	SAE	0	1 (6.2%)	1 (2.6%)	2 (5.4%)
	Drug-related AE	0	2 (12.5%)	2 (5.3%)	4 (10.8%)
	g3-5	0	5 (31.2%)	4 (10.5%)	4 (10.8%)
**Dehydration**					
	AE	0	0	2 (5.3%)	1 (2.7%)
	SAE	0	0	1 (2.6%)	0
	Drug-related AE	0	0	0	1 (2.7%)
	g3-5	0	0	1 (2.6%)	0
**Rash**					
	AE	1 (25%)	9 (56.2%)	24 (63.1%)	28 (75.7%)
	SAE*	3 (75%)	14 (87.5%)	28 (73.7%)	25 (67.6%)
	Drug-related AE	1 (25%)	8 (50%)	19 (50%)	20 (54%)
	g3-5	1 (25%)	0	2 (5.26%)	2 (5.4%)
**Acneiform dermatitis**					
	AE	2 (50%)	8 (50%)	11 (28.9%)	4 (10.8%)
	SAE*	3 (75%)	14 (87.5%)	28 (73.7%)	25 (67.6%)
	Drug-related AE	2 (50%)	6 (37.5%)	9 (23.7%)	3 (8.1%)
	g3-5	0	0	1 (2.6%)	0
**Paronychia**					
	AE	1 (25%)	1 (6.2%)	8 (21.05%)	6 (16.2%)
	SAE	0	0	0	0
	Drug-related AE	0	1 (6.2%)	5 (13.1%)	4 (10.8%)
	g3-5	0	0	0	0
**Related deaths**		0	0	1 (2.6%)**	0
Treatment discontinuations***		0	1 (6.25%)	2 (5.26%)	1 (2.7%)

N, number of patients; AE, adverse event; SAE serious adverse event; g 3-5, grade 3 to 5 according to National Cancer Institute Common Terminology Criteria for Adverse Events.

*The information on SAE related to the skin is collected together.

**One patient presented with Interstitial Lung Disease and died despite discontinuing erlotinib.

***Includes only discontinuations due to drug-related SAEs.

Erlotinib-related toxicities in the phase II trial included skin toxicity (reported as rash or acneiform dermatitis) in 35 (92.1%) patients in the control arm and 32 (86.4%) patients in the experimental arm, asthenia in 14 (36.8%) and 14 (37.8%) patients, dehydration in 2 (5.3%) and 1 (2.7%), and diarrhea in 27 (71%) and 30 (81.1%), respectively. Hyperglycemia was seen in 5 (13.1%) patients in the control arm and 7 (18.9%) in the experimental arm. Grade 3-5 hyperglycemia was observed in 4 (10.52%) and 4 (10.81%) patients, respectively. Although grade 3 to 5 adverse events are reported together, no grade 5 hyperglycemia was observed. One death, probably related to interstitial lung disease, was reported in the erlotinib arm (2.63%) (Table 2).

### Efficacy

No significant differences were observed in PFS or OS between the two treatment arms in the phase II trial. PFS was 1.6 months in the control arm and 2.5 months in the experimental arm (hazard ratio [HR]: 0.86, 95% CI: 0.47-1.57; *p* =0.268). OS was 10.2 months in the control arm and 6.6 months in the experimental arm (HR: 1.80, 95% CI: 0.87-3.72; *p* =0.946].

No significant difference in overall response rate was observed between the two arms. Three partial responses (PR) were observed in the control arm (7.9%) and one (2.8%) in the experimental arm, and there were no complete responses in either arm. All four patients with PR had adenocarcinomas. Twenty-one (55.3%) patients in the control arm and 21 (56.7%) in the experimental arm had stable disease (SD) as best response. Stable disease between 3 and 12 months was attained by seven patients in each arm (18.4% and 18.9%, respectively), and SD lasted longer than 12 months in three and two patients, respectively (Table 3).

**Table 3:** **Summary of outcomes in phase II trial**

Outcome	Erlotinib	Dalotuzumab 10 mg/kg plus erlotinib	
	N = 38	N = 37	
**Response**			Difference in rates: -0.052 (95% CI: -0.069-0.185); *p* = 0.317
Partial response	3 (7.9%)	1 (2.7%)	
Complete response	0	0	
Overall response	3 (7.9%)	1 (2.7%)	
Not evaluable	1 (2.6%)	4 (10.8%)	
Progressive disease	14 (36.8%)	11 (29.7%)	
Stable disease	21 (55.3%)	21 (56.7%)	
Stable disease ≤3 months	11 (28.9%)	10 (27%)	
Stable disease 4-12 months	7 (18.42%)	7 (18.9%)	
Stable disease >12 months	3 (7.9%)	2 (5.4%)	
**Progression-free survival (months)**	1.6	2.5	HR, 0.86 (95% CI: 0.47-1.57); *p* = 0.268
**Overall survival (months)**	10.2	6.6	HR, 1.80 (95% CI: 0.87-3.72); *p* = 0.946

HR = hazard ratio.

Fourteen patients in the erlotinib arm crossed over to the experimental arm at the time of disease progression. The best response for this subgroup of patients was SD in four patients (lasting 15 months in one patient and less than three months in three patients). At the time of the first evaluation after crossover, six patients had progressed and no response assessment was done in four patients. None of the patients who crossed over harbored *EGFR* or *KRAS* mutations.

## Discussion

Although the present study has confirmed the general safety and tolerability of the combination of erlotinib plus dalotuzumab in patients with advanced NSCLC, no benefit was observed in terms of efficacy. In fact, the RR for the combination of erlotinib and dalotuzumab was lower than the 8-10% obtained with standard therapies in recurrent advanced NSCLC, including not only chemotherapy but also EGFR tyrosine kinase inhibitors (TKIs) such as gefitinib and erlotinib [10, 11]. Even though the sample size was small, the study was powered to detect clinically significant differences in PFS.

As expected from previous dose-escalation studies, toxicity was mild and mainly related to erlotinib [12–14].

The major concern regarding IGF1R inhibition is the risk of hyperglycemia. In a previous trial of figitumumab, 15% of patients previously had been diagnosed with diabetes, and grade 3-5 hyperglycemia rates were almost 20% in both arms of the trial [15]. In our study, less than 20% of the patients in both arms had hyperglycemia (grade 3-4 in 10% in each arm), and in all cases, it was manageable with insulin. Although grade 3 and 4 hyperglycemia was recorded, it was not considered a DLT since according to the protocol specifications the event was controlled within the first 48 hours. Moreover, hyperglycemia was well controlled in the current study in patients with a previous diagnosis of diabetes mellitus who were receiving treatment at enrollment.

The cumulative experience of using MoAbs directed against IGF1R in combination with different therapies in unselected NSCLC patients raises the question of why promising evidence in the laboratory has failed repeatedly when translated into the clinical setting. One reason could be the excessive toxicity of the combinations. For example, when anti-IGF1R MoAbs were combined with chemotherapy or erlotinib, toxicity may well have prevented the optimum synergistic effect. Conversely, and similar to our findings, the toxicity profile was quite acceptable with erlotinib plus R1507 [16]. In fact, the majority of side effects were related to erlotinib, and the addition of the anti-IGF1R MoAb did not significantly increase the risk of toxicity.

A second explanation for the failure of dual IGF1R and EGFR inhibition may lie in the influence of *KRAS* or *EGFR* mutations. Patients with *KRAS* mutations had a higher 12-week PFS rate than those with wild-type *KRAS* (36% vs. 0%) when treated with erlotinib plus R1507 [16]. In the present study, all five patients with *KRAS* mutations had progressive disease. *EGFR* mutations did not correlate with outcome to erlotinib plus R1507 [16]. In the present study, only one of the four patients with *EGFR* mutations was allocated to the experimental arm. Although this patient had stable disease lasting seven months, it is impossible to draw any conclusions from this individual case.

## Conclusion

In conclusion, the present study has shown that although the combination of dalotuzumab plus erlotinib is generally tolerable, it does not confer greater benefit than erlotinib alone in advanced NSCLC patients who are EGFR-TKI naïve. The cumulative results of this and other studies of dual IGF1R and EGFR inhibition indicate that further investigation of such combinations is not warranted in unselected NSCLC patients.

### Consent

Written inform consent form included the patient authorization for publication of data obtained from the present trial.

## Author’s information

Teresa Moran: Presented in part at the 100th Annual Meeting of the American Association for Cancer Research (April 2009) and at the IASLC 14th World Conference on Lung Cancer (July 2011).
